# P65 mediated UBR4 in exosomes derived from menstrual blood stromal cells to reduce endometrial fibrosis by regulating YAP Ubiquitination

**DOI:** 10.1186/s12951-023-02070-3

**Published:** 2023-08-29

**Authors:** Jiarui Qi, Xudong Zhang, Siwen Zhang, Shanshan Wu, Yimeng Lu, Shuyu Li, Pingping Li, Jichun Tan

**Affiliations:** 1 Center of Reproductive Medicine, Department of Obstetrics and Gynecology, Shengjing Hospital of China Medical University, No. 39 Huaxiang Road, Tiexi District, Shenyang, 110022 China; 2 Key Laboratory of Reproductive Dysfunction Disease and Fertility Remodeling of Liaoning Province, No. 39 Huaxiang Road, Tiexi District, Shenyang, 110022 China; 3 Key Laboratory of Reproductive and Genetic Medicine (China Medical University), National Health Commission, Shenyang, China

**Keywords:** MenSCs, Exosomes, Endometrial fibrosis, ubr4, Ubiquitination

## Abstract

**Background:**

Intrauterine adhesion (IUA) is a recurrent and refractory reproductive dysfunction disorder for which menstrual blood-derived stromal cells (MenSCs) might be a promising intervention. We reported that administration of MenSCs-derived exosomes (MenSCs-EXO) could achieve similar therapeutic effects to MenSCs transplantation, including alleviating endometrial fibrosis and improving fertility in IUA rats. The mass spectrometry sequencing result suggested that UBR4, a member of the proteasome family, was abundantly enriched in MenSCs-EXO. This study aimed to investigate the key role of UBR4 in MenSCs-EXO for the treatment of IUA and the specific molecular mechanism.

**Results:**

UBR4 was lowly expressed in the endometrial stromal cells (EndoSCs) of IUA patients. MenSCs-EXO treatment could restore the morphology of IUA endometrium, reduce the extent of fibrosis, and promote endometrial and vascular proliferation. Knockdown of UBR4 in MenSCs did not affect the characteristics of exosomes but attenuated the therapeutic effect of exosomes. UBR4 in MenSCs-EXO could alleviate endometrial fibrosis by boosting YAP ubiquitination degradation and promoting YAP nuclear-cytoplasmic translocation. Moreover, P65 could bind to the UBR4 promoter region to transcriptionally promote the expression level of UBR4 in MenSCs.

**Conclusion:**

Our study clarified that MenSCs-EXO ameliorated endometrial fibrosis in IUA primarily by affecting YAP activity mediated through UBR4, while inflammatory signaling P65 may affect UBR4 expression in MenSCs to enhance MenSCs-EXO therapeutic effects. This revealed a novel mechanism for the treatment of IUA with MenSCs-EXO, proposing a potential option for the clinical treatment of endometrial injury.

## Background

Intrauterine adhesions (IUA) refer to a series of syndromes caused by severe damage to the basal layer of endometrium, forming a fibrotic scar, leading to partial or complete uterine cavity atresia [1]. The common causes of IUA are frequent uterine cavity manipulation, infection, ischemia, etc., and the main manifestations are amenorrhea, reduced menstrual volume, recurrent abortion, infertility, and placental abnormalities [2]. IUA seriously impacts female reproductive function and conventional therapies such as hysteroscopic adhesiolysis and hormone supplementation have poor efficacy in the treatment of severe IUA patients [3]. Therefore, it is urgent to find a novel treatment option for IUA.

In recent years, several clinical trials have confirmed the effectiveness of mesenchymal stem cells (MSCs) derived from bone marrow and umbilical cord in the treatment of severe IUA [4–6]. Our previous study also found that autologous transplantation of menstrual blood-derived stromal cells (MenSCs) could increase endometrial thickness and pregnancy rate in patients with severe IUA [7]. Recent studies further suggested that exosomes might be key paracrine mediators of the therapeutic effect of MSCs and could effectively avoid the side effects of cell therapy [8]. Exosomes, as extracellular vesicles with a particle size of 30-150 nm carrying a large number of biomolecules such as RNA, proteins, and miRNAs encapsulated by lipid bilayers can mediate cell-to-cell communication, which has great potential for the clinical treatment of a wide range of diseases [9]. In recent years, exosomes have been demonstrated as a potential therapeutic approach for the improvement of reproductive disorders such as premature ovarian insufficiency (POI), polycystic ovary syndrome (PCOS), and declined fertility related to aging [10–12]. Specifically, exosomes could play an important role in tissue injury repair by promoting tissue proliferation and vascular regeneration, and inhibiting inflammation of damaged tissues [13]. It has been reported that exosomes derived from bone marrow MSCs and adipose MSCs could alleviate endometrial fibrosis in IUA animals [14–16]. Our previous study also further confirmed that exosomes derived from MenSCs (MenSCs-EXO) had a similar therapeutic effect as MenSCs, which could effectively and safely remodel the morphology of the uterine cavity and the function of the endometrium [17, 18]. Therefore, we aimed to further explore the key molecules in MenSCs-EXO for the treatment of IUA as well as the specific molecular mechanisms for repairing the damaged endometrium.

Yes-associated protein (YAP) is a key molecule in the Hippo signaling pathway, which is related to cell proliferation and apoptosis, tissue repair, and regeneration [19]. During classical Hippo pathway activation, MST1/2 kinase phosphorylation activates LATS1/2, which in turn inhibits YAP/TAZ nuclear translocation by phosphorylating YAP/TAZ. When the Hippo pathway is inhibited, YAP/TAZ translocates into the nucleus and binds to the transcription factor TEAD to activate the transcription of target genes, thereby regulating the function of target cells [20, 21]. YAP has been reported to be associated with fibrotic processes in various organs such as the urinary tract and liver, YAP has also played an important role in scar-free repair of the endometrium during menstruation [22–24]. An in vitro study showed that MenSCs inhibited fibroblast activation and attenuated endometrial fibrosis by activating the Hippo pathway through paracrine effects [25]. Our in vitro experiment proposed that MenSCs-EXO might inhibit fibrosis of endometrial stromal cells (EndoSCs) in IUA patients (IUA-EndoSCs) by promoting the ubiquitination and subsequent degradation of YAP. However, the molecular mechanism by which MenSCs-EXO regulate YAP expression in IUA-EndoSCs remains unclear.

Dysregulation of ubiquitin-mediated protein degradation can cause a variety of diseases, including cancer, neurodegenerative diseases, and tissue fibrosis [26]. E3 ubiquitin ligase, which could especially regulate target gene protein expression, emerged as a potential therapeutic target in recent years [27]. E3 ubiquitin ligase β-TRCP in exosomes of human umbilical cord MSCs was demonstrated to attenuate renal fibrosis by ubiquitinating degradation of YAP [28]. Our previous mass spectrometry of MenSCs-EXO also showed that the proteasome system was highly enriched in MenSCs-EXO, and UBR4 was the most abundant expression of E3s. UBR4 has been confirmed to be involved in many physiological and pathological processes through the ubiquitin-proteasome pathway. UBR4 was involved in early endosomal biosynthesis and early embryonic nervous system development [29, 30], and could also regulate muscle fiber size and muscle protein mass during aging [31]. Moreover, UBR4 was associated with enhanced invasiveness of gastric cancer cells [32]. Given these, we hypothesized that UBR4 in MenSCs-EXO attenuated endometrial fibrosis by contributing to the ubiquitinated degradation of YAP in the endometrium.

P65 is a subunit of the transcription factor NF-κB, which plays an important role in the regulation of tissue damage and repair [33, 34] and can regulate the endometrial periodic changes and endometrial angiogenesis [35, 36]. Activation of the NF-κB signaling pathway in hematopoietic stem cells can enhance their survival and function [37]. Our previous results have also shown that both platelet-rich plasma and platelet-derived growth factor-BB improved the biological function and antifibrotic function of MenSCs by activating the NF-κB signaling pathway, but the underlying mechanism of the over-expression of P65 enhancing MenSCs function is still unclear. Bioinformatics analysis predicted that the UBR4 promoter had a conserved binding site of P65, therefore we speculated that P65 in the inflammatory microenvironment of IUA might mediate the remodel of endometrial fibrosis through transcriptional regulation of UBR4 expression in MenSCs as well as MenSCs-EXO.

In this study, we aimed to investigate the key role of UBR4 in MenSCs-EXO concerning the amelioration of endometrial fibrosis, and the molecular mechanisms involved in the regulation of YAP activity. Meanwhile, we further clarified whether P65 could regulate the expression level of UBR4 and its molecular mechanism. Our study explained the molecular and signaling pathways involved in the inhibition of endometrial fibrosis by MenSCs-EXO and provided a theoretical basis for the optimization of MenSCs-EXO therapy for IUA patients.

## Results

### UBR4 was lowly expressed in IUA-EndoSCs

**Fig. 1:**
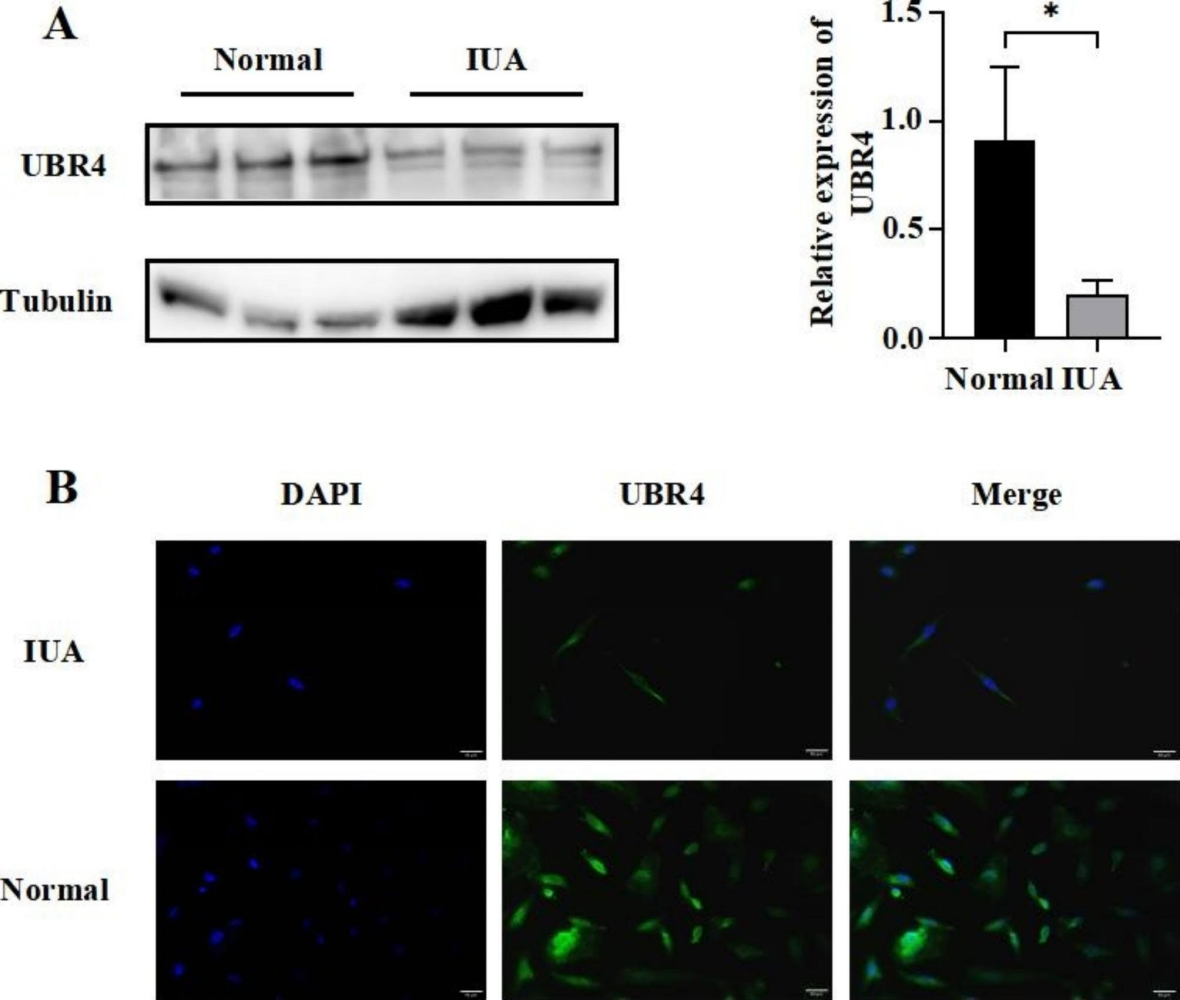
UBR4 was lowly expressed in IUA-EndoSCs. (**A**) Western blot of UBR4; (**B**) Immunofluorescence assays of UBR4 (green) and DAPI was used to stain nuclei (Blue). Data were mean ± SD, *P < 0.05, **P < 0.01, ***P < 0.001 for Student’s t-test

We examined the expression of UBR4 in IUA-EndoSCs, and we found that the expression of UBR4 in IUA-EndoSCs was significantly reduced compared with EndoSCs of normal people (*P* < 0.05) (Fig. 1A). Immunofluorescence results were consistent with Western Blot results, showing that UBR4 expression in IUA-EndoSCs was decreased in both the nucleus and cytoplasm compared with normal EndoSCs (Fig. 1B).

### UBR4 concentration was significantly reduced in EXO^UBR4−^ but exosome characteristics remained unchanged

**Fig. 2:**
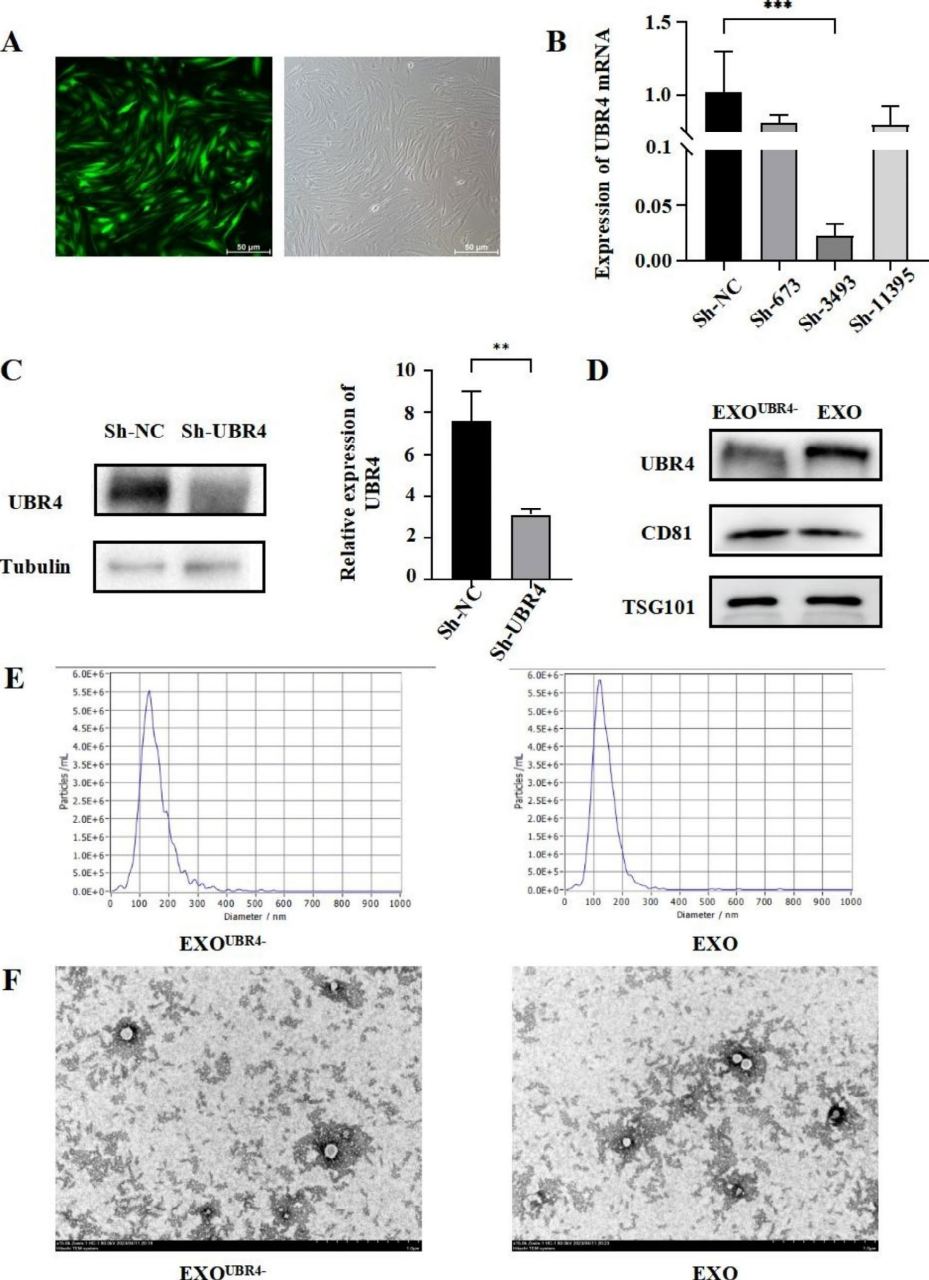
UBR4 concentration was significantly reduced in EXO^UBR4−^ but exosome characteristics remained unchanged. (**A**) Transfection of ADV-Sh-UBR4 into MenSCs; (**B**) Detection of UBR4 mRNA levels by RT-PCR; (**C**) Western blot of UBR4 of MenSCs; (**D**) Western blot of UBR4, CD81, and TSG101 of EXO^UBR4−^ and EXO; (**E**) Size distribution of EXO^UBR4−^ and EXO examined by NTA; (**F**) Morphology of MenSCs-EXO observed by TEM (scale bar = 1 μm). Data were mean ± SD, *P < 0.05, **P < 0.01, ***P < 0.001 for Student’s t-test

Our preliminary results confirmed that MenSCs-EXO could reduce endometrial fibrosis and improve fertility in IUA rats, and the preliminary mass spectrometry results suggested that UBR4 was highly enriched in exosomes. To further investigate the mechanism of UBR4 in the treatment of IUA by MenSCs-EXO, we transfected ADV-Sh-UBR4 to MenSCs to reduce the expression of UBR4 (Fig. 2A). We constructed three lentiviruses with three different sequences and detected the knock-down efficiency of each lentivirus by RT-PCR. We found that the knock-down efficiency of Sh-3493 could reach more than 90%, while the knock-down efficiency of Sh-673 and Sh-11395 was not ideal (Fig. 2B). Western Blot was used to detect the expression of UBR4 in MenSCs transfected with Sh-3493, and the results showed that Sh-3493 could significantly reduce the protein level of UBR4 compared to Sh-NC (*P* < 0.01) **(Fig. 2C)**. Therefore, we decided to use Sh-3493 to complete the subsequent experiments. We next collected exosomes from UBR4-knockdown MenSCs. We found that the expression of UBR4 was significantly lower in the EXO^UBR4−^ compared with normal exosomes, but EXO^UBR4−^ and EXO had the same particle size and surface markers, and transmission electron microscopy showed no difference in the morphology of the two types of exosomes and the protein concentration between the two types of exosomes had no statistical difference**(Fig. 2D-F)**. These results suggested that transfection of lentiviruses with knockdown UBR4 into MenSCs did not affect the characteristics of exosomes secreted by MenSCs, but only decrease the expression of UBR4 in exosomes.

### Reducing the content of UBR4 in MenSCs-EXO would alleviate its therapeutic effect on IUA rats

**Fig. 3:**
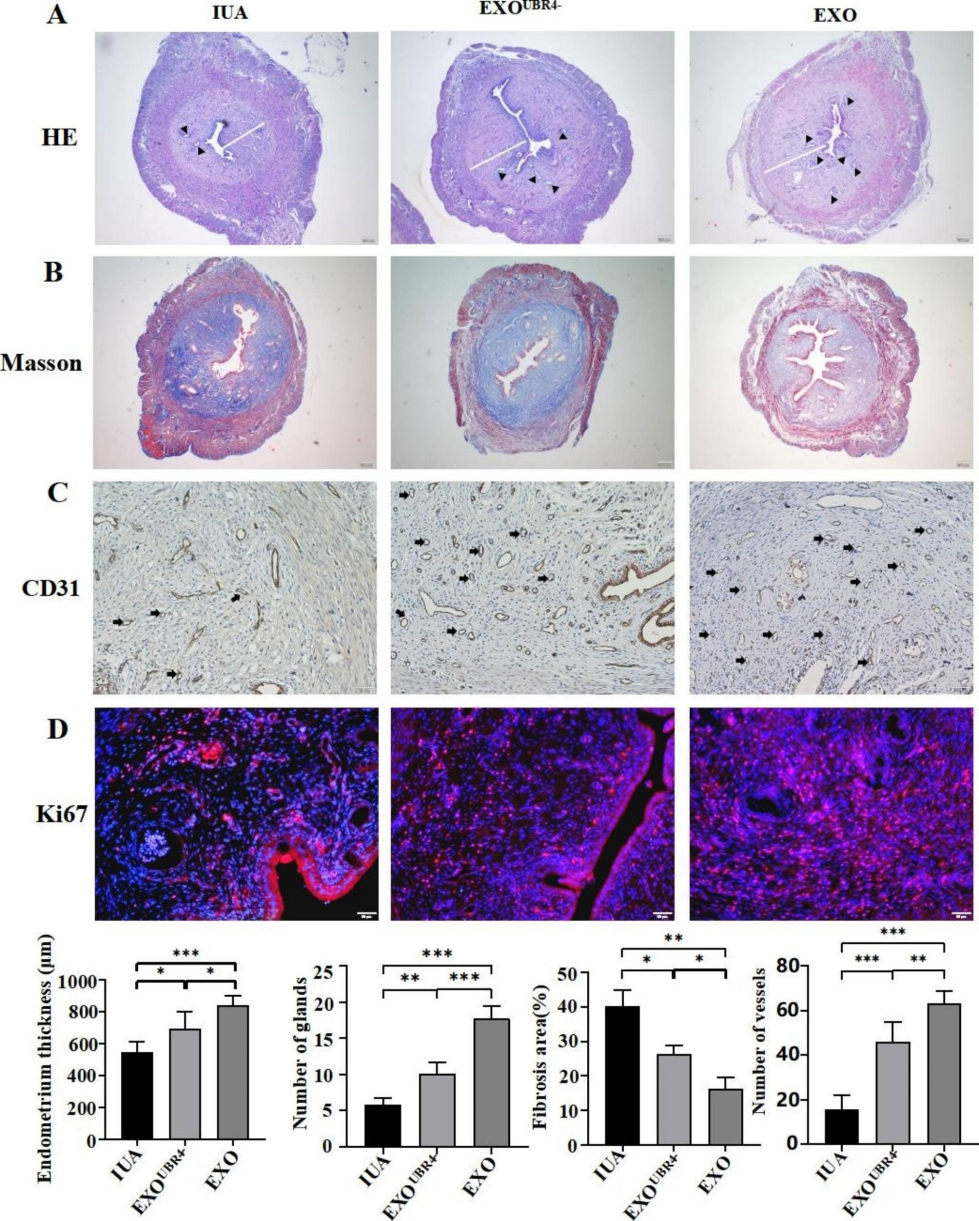
Reducing the content of UBR4 in MenSCs-EXO would alleviate its therapeutic effect on IUA rats. (**A**) H&E staining of uterine tissue (Scale bar = 200 μm) (The white line indicates endometrial thickness, the black triangles indicate glands); (**B**) Masson staining of uterine tissue (Scale bar = 200 μm); (**C**) Immunohistochemical staining of CD31 (Scale bar = 50 μm) (The black arrows indicate neovascularization); (**D**) Immunofluorescence assays of Ki67 (red) and DAPI was used to stain nuclei (Blue). (Scale bar = 50 μm). Data were mean ± SD, *P < 0.05, **P < 0.01, ***P < 0.001 for One Way ANOVA

To further explore the differences in the treatment effects between EXO^UBR4−^ and EXO, we used these two kinds of exosomes to treat IUA model rats. HE staining was used to observe the endometrial morphology, endometrial thickness, and number of glands. The HE staining results showed that treatment with both EXO^UBR4−^ and EXO increased the endometrial thickness (*P* < 0.05, *P* < 0.001) and number of glands (*P* < 0.01, *P* < 0.001) compared with the IUA group, but the endometrial thickness in the EXO^UBR4−^ treated group was lower than that in the EXO treated group (*P* < 0.001) and number of glands in the EXO^UBR4−^ treated group was less than that in the EXO treated group (*P* < 0.05) **(**Fig. 3A**)**. Masson staining results showed that treatment with both EXO^UBR4−^ and EXO could reduce the area of endometrial fibrosis in IUA rats compared with the IUA group,(*P* < 0.05, *P* < 0.01) but the area of endometrial fibrosis in the EXO^UBR4−^ treated group was larger than that in the EXO treated group (*P* < 0.05) **(**Fig. 3B**)**. Next, we observed endometrial neovascularization and proliferation in IUA rats. The results showed that the number of neovascularization in both EXO^UBR4−^ and EXO treated groups was higher than that in the IUA group, (*P* < 0.001, *P* < 0.01) but the number of neovascularization in the EXO treated group was higher than that in the EXO^UBR4−^treated group (*P* < 0.001)**(**Fig. 3C**)**. Both EXO^UBR4−^ and EXO treatment could promote endometrial proliferation in IUA rats, but the pro-proliferative ability of EXO^UBR4−^ was weaker than that of EXO **(**Fig. 3D**)**.

### Reducing the content of UBR4 in MenSCs-EXO would alleviate its anti-fibrotic effect

**Fig. 4:**
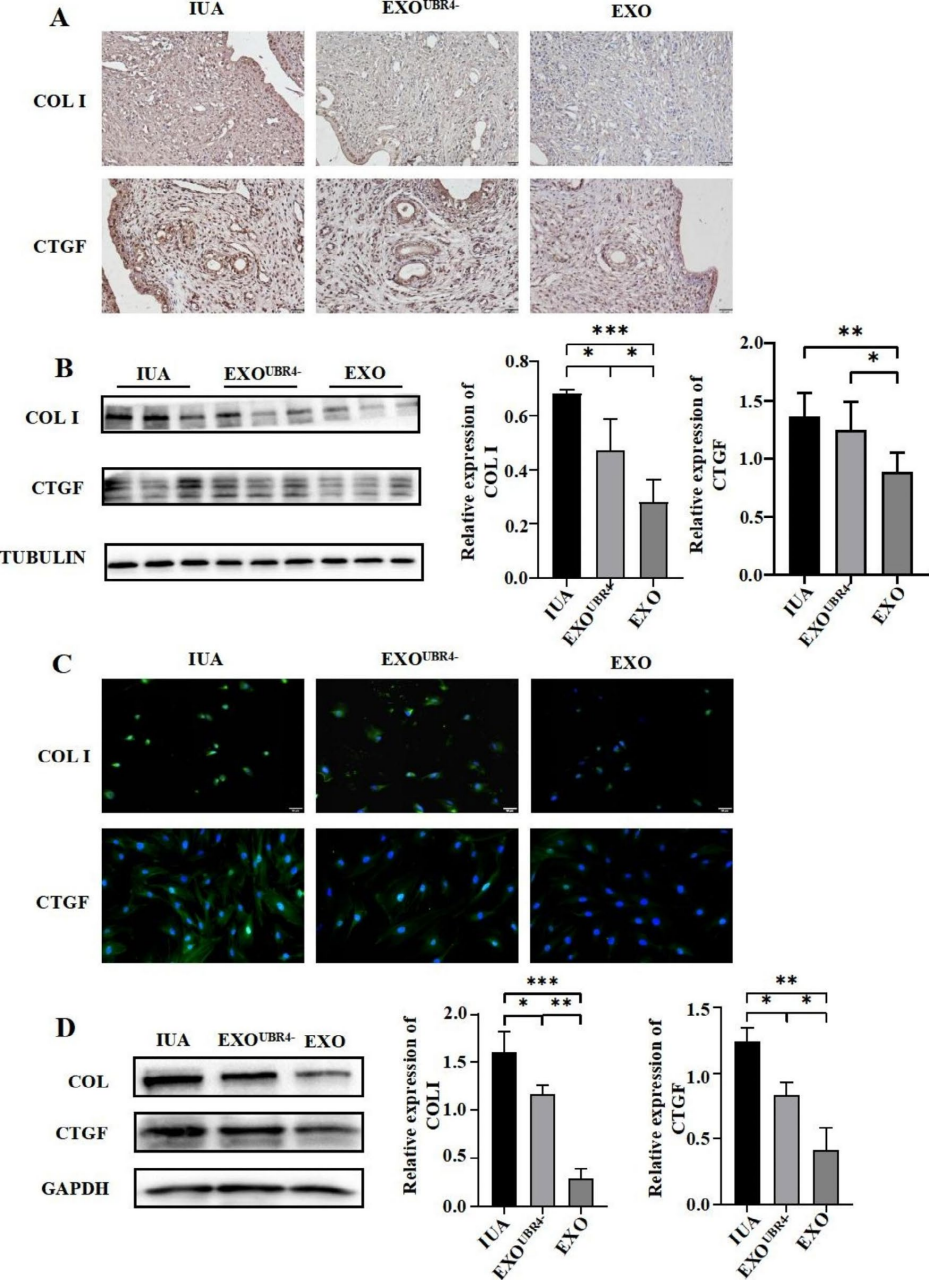
Reducing the content of UBR4 in MenSCs-EXO would alleviate its anti-fibrotic effect. The IUA model rats and IUA-EndoSCs were treated with EXO and EXO^UBR4−^, respectively. (**A**) Immunohistochemical staining of COL I and CTGF in the endometrium of IUA rats (Scale bar = 20 μm); (**B**) Western blot (left) and relative quantification (right) of COL I and CTGF in rat endometrium; (**C**) Immunofluorescence assays of COL I and CTGF (green) and DAPI was used to stain nuclei (Blue). (Scale bar = 50 μm); (**D**) Western blot (left) and relative quantification (right) of COL I and CTGF in IUA-EndoSCs before and after treatment. Data were mean ± SD, *P < 0.05, **P < 0.01, ***P < 0.001 for One Way ANOVA

Next, we evaluated the expression of fibrosis-related indicators (COL I and CTGF) in the endometrium of rats in the IUA group, EXO^UBR4−^ group, and EXO group, respectively. The results of immunohistochemistry and Western Blot showed that COL I and CTGF were both highly expressed in the endometrium of IUA rats, and the expression levels of COL I were significantly lower in both EXO^UBR4−^ and EXO groups compared with the IUA group (*P* < 0.05, *P* < 0.001), but the expression level of COL I was more significantly decreased in the EXO group (*P* < 0.05) (Fig. 4A **and B**). The expression of CTGF had no significant difference in the IUA and EXO^UBR4−^ groups but was significantly lower in the EXO group (*P* < 0.01) (Fig. 4A **and B**). Meanwhile, we also examined the expression levels of COL I and CTGF in IUA-EndoSCs, and we found that COL I and CTGF were both highly expressed in the IUA group, and both EXO^UBR4−^ and EXO treatment could decrease the expression of COL I (*P* < 0.05, *P* < 0.001) and CTGF (*P* < 0.05, *P* < 0.01), but the expression levels of COL I and CTGF decreased more significantly in the EXO treatment group (*P* < 0.01, *P* < 0.05) (Fig. 4C **and D**), which was similar to the results in vivo.

### UBR4 inhibited endometrial fibrosis through ubiquitinated degradation of YAP

**Fig. 5:**
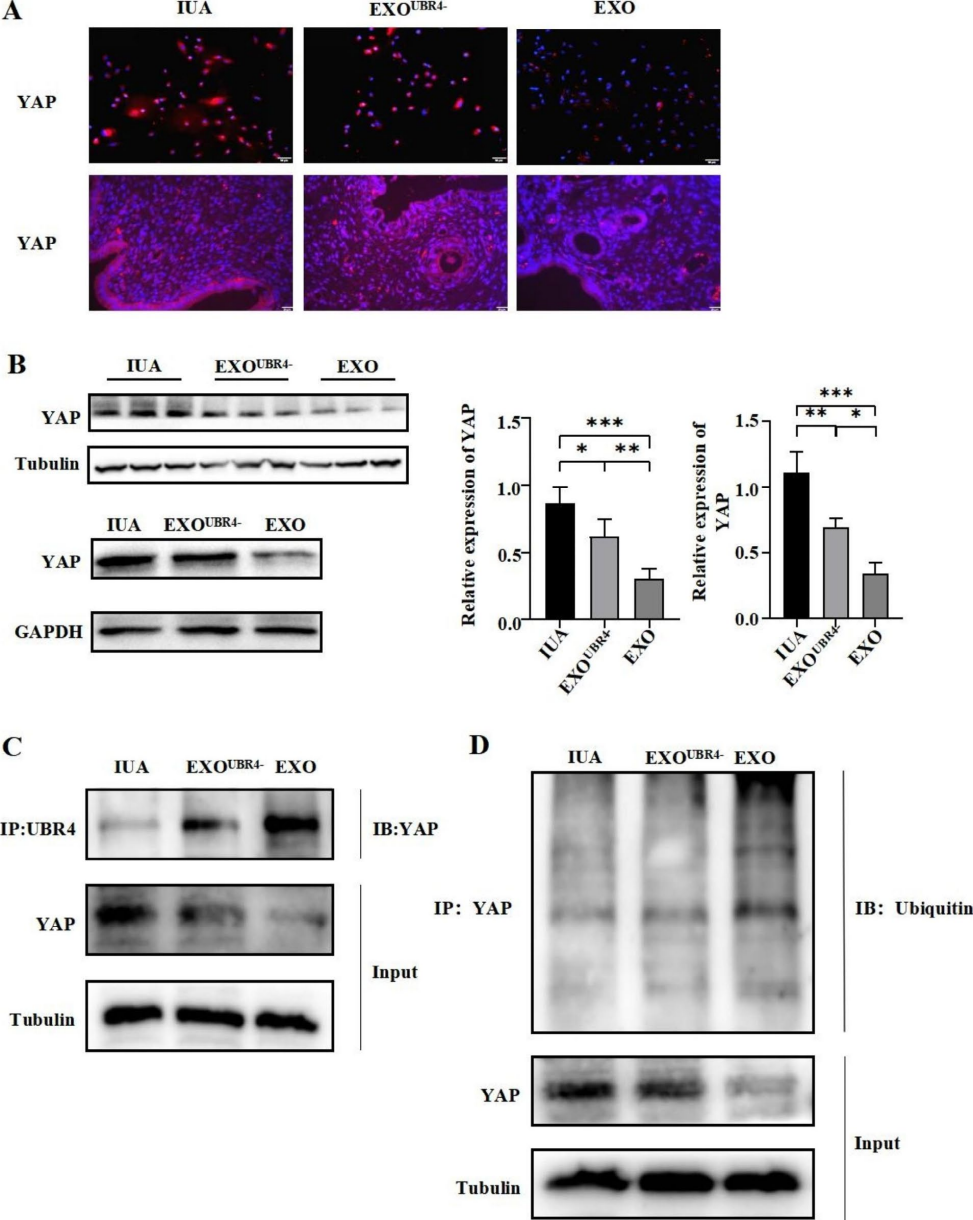
UBR4 inhibited endometrial fibrosis through ubiquitinated degradation of YAP. The IUA model rats and IUA-EndoSCs were treated with EXO and EXO^UBR4−^, respectively. (**A**) Immunofluorescence assays of YAP (red) and DAPI was used to stain nuclei (Blue) in IUA-EndoSCs (upper) (Scale bar = 50 μm)and endometrium of IUA rats (lower). (Scale bar = 20 μm); (**B**) Western blot (left) and relative quantification (right) of YAP in the endometrium of IUA rats (upper) and IUA-EndoSCs (lower); (**C**) CO-IP assay of UBR4 and YAP were performed to investigate the interactions between UBR4 and YAP in IUA-EndoSCs; (**D**) Immunoprecipitation to determine YAP ubiquitination in IUA-EndoSCs before and after treatment. Data were mean ± SD, *P < 0.05, **P < 0.01, ***P < 0.001 for One Way ANOVA

Next, we investigated the underlying molecular mechanism of UBR4 as the key factor in the exosome treatment of IUA. Combined with our previous findings that MenSCs-EXO treatment decreased the expression of YAP in the endometrium of IUA, we investigated whether UBR4 in MenSCs-EXO reduced the fibrosis level of IUA endometrium by regulating the expression level of YAP. First, we treated IUA rats with EXO^UBR4−^ and EXO respectively, and the immunofluorescence results showed that the expression level of YAP decreased after treatment with EXO^UBR4−^, but not significantly. In contrast, the expression level of YAP was significantly decreased after treatment with EXO, and the expression level of YAP was more significantly decreased in the nucleus than in the cytoplasm **(**Fig. 5A). Western Blot results showed that both EXO^UBR4−^ and EXO treatment could decrease YAP expression in the endometrium compared to the IUA group (*P* < 0.05, *P* < 0.001), but the decrease was greater in the EXO group than in the EXO^UBR4−^ group (*P* < 0.01) (Fig. 5B). Similarly, we treated IUA-EndoSCs with EXO^UBR4−^ and EXO respectively in vitro. Immunofluorescence and Western Blot results showed that both EXO^UBR4−^ and EXO could effectively reduce the expression level of YAP (*P* < 0.01, *P* < 0.001), but the reduction of YAP expression was higher in the EXO group than in the EXO^UBR4−^ group (*P* < 0.05) (Fig. 5A and B). To further investigate the molecular mechanism of UBR4 regulation of YAP expression, we examined the ubiquitination level of YAP in the EndoSCs of IUA patients treated with EXO^UBR4−^ and EXO. Co-immunoprecipitation assay results showed that UBR4 and YAP could specifically bind in EndoSCs (Fig. 5C), and the ubiquitination level of YAP in EXO^UBR4−^ and EXO groups was higher than that in the IUA group and the ubiquitination level of YAP was higher in the EXO group than that in the EXO^UBR4−^ group (Fig. 5D).

### P65 transcriptional activation promoted UBR4 expression in MenSCs

**Fig. 6:**
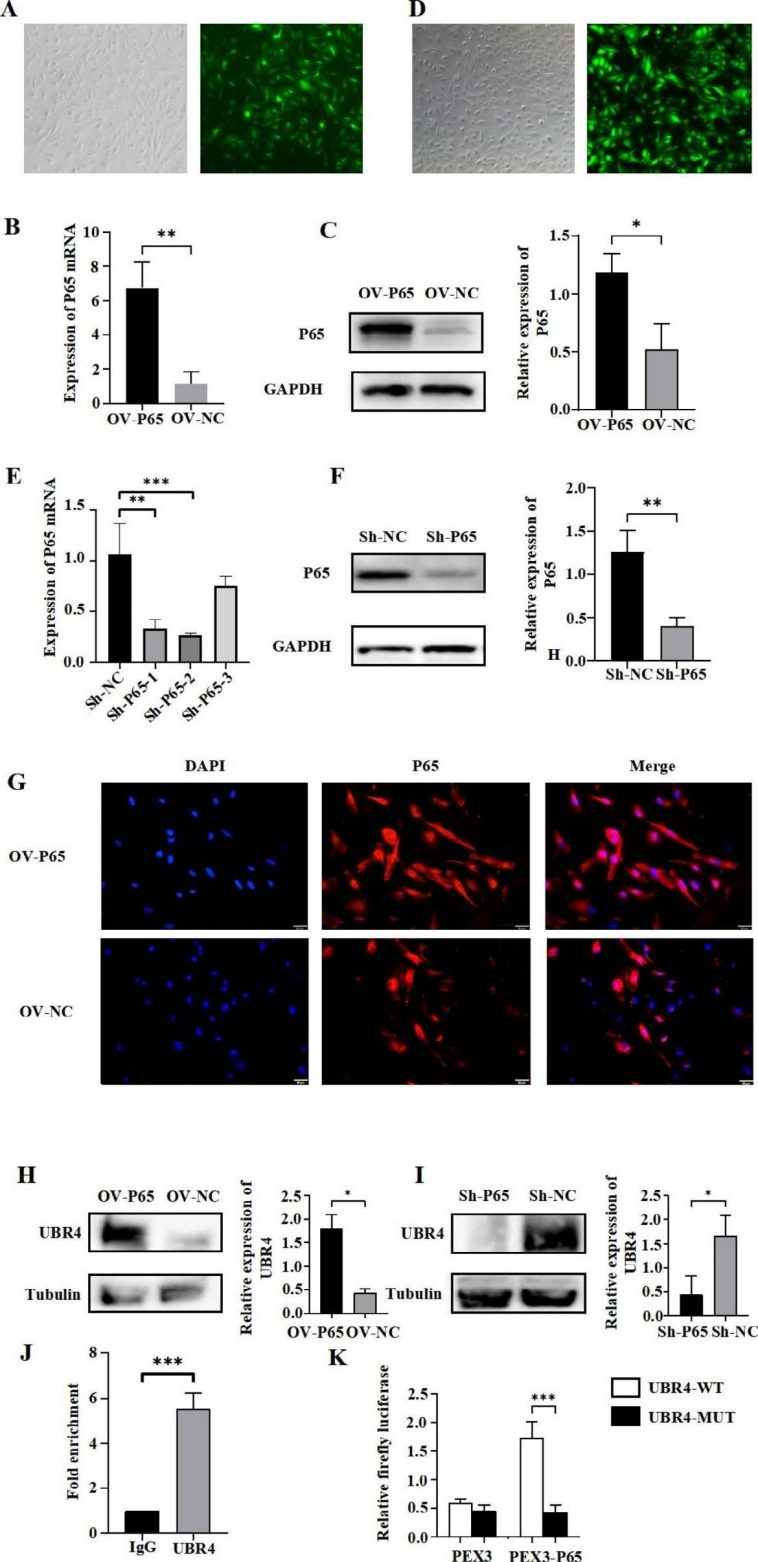
P65 transcriptional activation promoted UBR4 expression in MenSCs. (**A**) Transfection of ADV-OV-P65 into MenSCs; (**B**) Detection of P65 mRNA levels by RT-PCR; (**C**) Western blot of P65 in MenSCs; (**D**) Transfection of ADV-Sh-P65 into MenSCs; (**E**) Detection of P65 mRNA levels by RT-PCR; (**F**) Western blot of P65 in MenSCs; (**G**) Immunofluorescence assays of P65 (red) and DAPI was used to stain nuclei (Blue) in MenSCs; (**H**) Western blot (left) and relative quantification (right) of UBR4 in MenSCs with or without overexpression of P65; (**I**) Western blot (left) and relative quantification (right) of UBR4 in MenSCs with or without knockdown of P65; (**J**) ChIP to evaluate P65 enrichment in the promoter region of UBR4 gene; (**K**) Dual-luciferase assay was performed to detect the activity and showed that UBR4 bond PEX3‐P65. Data were mean ± SD, *P < 0.05, **P < 0.01, ***P < 0.001 for One Way ANOVA

Although UBR4 has been identified as a key factor in MenSCs-EXO to promote endometrial repair in IUA, we still do not know what factors regulate the expression of UBR4 in menstrual blood stem cells. Our previous results showed that over-expression of transcription factor P65 could enhance the biological function of menstrual blood stem cells and improve the antifibrotic effect of MenSCs-EXO. Therefore, we hypothesized that P65 might enhance the antifibrotic therapeutic effect of MenSCs-EXO by regulating the expression of UBR4. To explore the transcriptional regulation relationship between P65 and UBR4, we first constructed P65 over-expressing lentivirus (OV-P65), then we transfected OV-P65 into MenSCs (Fig. 6A). RT-PCR and Western Blot results showed that the expression level of P65 was significantly increased after OV-P65 transfection (*P* < 0.01, *P* < 0.05) (Fig. 6B and C). Moreover, we found that P65 was transferred from the cytoplasm to the nucleus after overexpression of P65 (Fig. 6G). Next, we also conducted P65 knockdown experiments (Fig. 6B). We constructed three kinds of lentivirus to knockdown P65 and detected the knockdown efficiency of three different sequences by RT-PCR. We found that both Sh-P65-1 and Sh-P65-2 could effectively reduce the expression of P65, but the knockdown efficiency of Sh-P65-2 was more obvious (*P* < 0.01, *P* < 0.001) (Fig. 6E). Furthermore, Western Blot was used to detect the expression of the protein level of P65 in MenSCs transfected with Sh-P65-2, and the results showed that Sh-P65-2 could significantly reduce the protein level of P65 (*P* < 0.01) (Fig. 6F). Therefore, we decided to use Sh-P65-2 to complete the subsequent experiments. To explore whether P65 can regulate the expression level of UBR4, we also detected the expression level of UBR4 in MenSCs over-expressing and knocking down P65 by Western Blot. Western Blot results showed that the expression level of UBR4 increased and decreased significantly with the increase and decrease of P65 (*P* < 0.05, *P* < 0.05) (Fig. 6H and I). Although P65 is related to the expression level of UBR4, to further explore the potential mechanism of P65 regulating the expression level of UBR4, we used ChIp to pull down the DNA fragment bound to P65 and then used RT-PCR to detect whether this DNA fragment contained the UBR4 promoter region. ChIP results showed that P65 could bind to the promoter region of UBR4 to regulate its transcription (*P* < 0.001) (Fig. 6J). Next, we predicted the specific binding site for P65 at the UBR4 promoter region, designed wild-type and mutant-type plasmids of the UBR4 promoter region, and co-transfected them with P65 plasmid or control plasmid PEX3 into 293T cells. The fluorescence intensity of each group was detected by dual luciferase assay, and the results showed that the fluorescence intensity of UBR4-WT + PEX3-P65 was higher than other groups (*P* < 0.001) (Fig. 6K). These results suggest that P65 can promote UBR4 expression by binding to the UBR4 promoter region.

## Discussion

This study demonstrated that UBR4, as an E3 ubiquitin ligase, was a key molecule in MenSCs-EXO that inhibited the fibrosis of EndoSCs through specific ubiquitin degradation of YAP in EndoSCs. Furthermore, the transcription factor P65 highly expressed in IUA endometrial inflammatory microenvironment can bind to the UBR4 promoter region to promote the transcription of UBR4 and increase the expression level of UBR4. Our results suggested that the P65-UBR4-YAP signaling pathway might be a key molecular pathway in the treatment of IUA endometrial fibrosis by MenSCs-EXO.

UBR4 is a member of the N-terminal recognition family, whose conservative UBR box domain recognizes the n-terminal residues of substrate proteins and acts as an E3 ubiquitin ligase to degrade the substrate proteins via the ubiquitin-proteasome pathway [38]. The ubiquitin-proteasome system, as the main pathway of intracellular protein degradation, can bind ubiquitin to the bottom protein through ubiquitin-activating enzyme E1-ubiquitin-conjugating enzyme E2-ubiquitin-ligase E3 signal transmission, in which E3 ubiquitin-ligase plays the most important role in substrate-specific recognition [39]. Current reports have shown that E3 ubiquitin ligase played an important role in the occurrence and development of fibrosis in various organs. Hirotaka Fukasawa et al. reported that in the fibrotic kidney, the enhancement of specific ubiquitin degradation of Smad7 by Smurf1 and Smurf2 played a pathogenic role in the progression of tubulointerstitial fibrosis [40]. Similarly, NEDD4 could participate in keloid formation by enhancing the proliferation and invasiveness of fibroblasts and up-regulating the expression of type I collagen [41]. Our results suggested that the expression of UBR4 in the endometrium of IUA patients was significantly reduced, indicating that the loss of UBR4 may be related to endometrial fibrosis. Exosomes, a classic delivery vehicle [42], have been shown to repair fibrotic endometrium in IUA rats [43]. A study by *Sung Tae Kim et al.* indicated that UBR4 could be detected in endosomal and microvesicle membrane structures [30]. Our sequencing results also found that UBR4 was highly enriched in MenSCs-EXO. And we found that although treatment of IUA rats with EXO^UBR4−^ could improve the morphology and structure of endometrium, improve endometrial proliferation and vascular regeneration to some extent, and reduce the percentage of fibrosis area and expression level of fibrosis index, the therapeutic utility of EXO^UBR4−^ was significantly lower than that of EXO. This suggested that UBR4 might be a key molecule in MenSCs-EXO to repair fibrosis endometrium of IUA.

In the study of ubiquitin, it is the focus and difficulty to determine the role of E3 ubiquitin ligase and its specific substrate [44]. In this study, we have demonstrated that UBR4 played a key role in the treatment of IUA by MenSCs-EXO. Next, we wanted to explore the substrates that could specifically bind to UBR4 and the underlying molecular mechanism of UBR4 exerting anti-fibrosis effects through the ubiquitination pathway. The Hippo pathway is highly conserved during evolution. A large amount of evidence has shown that the Hippo pathway plays an important regulatory role in the process of fibrosis of the liver and kidney, which are derived from mesoderm as the same as the uterus [45, 46] and an in vitro study had shown that MenSCs could activate the Hippo pathway in EndoSCs through paracrine to inhibit the activation of fibroblasts and reduce TGF-β1-induced EndoSCs fibrosis [25]. Our previous results also found that MenSCs-EXO treatment decreased the expression level of YAP in the endometrium of IUA. Therefore, we speculated that YAP might be a ubiquitinated substrate of UBR4, and UBR4 could reduce the expression level of YAP through the ubiquitinated degradation of YAP. Our results revealed that YAP expression was significantly decreased when treating the endometrium of IUA rats and IUA-EndoSCs with EXO, however, the extent of reduction in YAP expression was not significant when treated with EXO^UBR4−^. These results suggested that UBR4 could significantly reduce the expression level of YAP.

Our Co-IP assay showed that UBR4 and YAP could bind to each other in EndoSCs.When treating IUA-EndoSCs with EXO^UBR4−^ and EXO, the YAP ubiquitination level in EXO^UBR4−^ treatment group was lower than that in the EXO treatment group. This indicated that when exogenous UBR4 supplementation increases, the YAP ubiquitination level in EndoSCs will also increase. These results suggested that UBR4 could increase the ubiquitination level of YAP in EndoSCs. In *Lu*’s study, the increased ubiquitination degradation of YAP could effectively inhibit liver fibrosis [47]. Our results showed that after EXO treatment, compared with the IUA group and EXO^UBR4−^ group, the expression level of YAP in the nucleus was significantly decreased. This suggested that MenSCs-EXO treatment could effectively prevent the nuclear translocation of YAP in EndoSCs. YAP is activated and translocated into the nucleus, where it binds to transcription factors such as TEADs to promote downstream transcription of pro-fibrosis genes such as CTGF and TGF-β [48]. CTGF can promote the deposition of extracellular matrix and is highly expressed in IUA endometrial tissue, which is closely related to the occurrence of IUA [49]. Multiple studies have shown that TGF-β can lead to endometrial fibrosis and IUA through the TGF-β/SMAD pathway [50, 51]. Therefore, UBR4 in MenSCs-EXO is likely to decrease the expression level of YAP by increasing the ubiquitin degradation of YAP in EndoSCs. Inhibition of YAP into the nucleus inhibited downstream fibrosis-promoting gene transcription to reduce fibrosis levels in IUA endometrium.

**Fig. 7:**
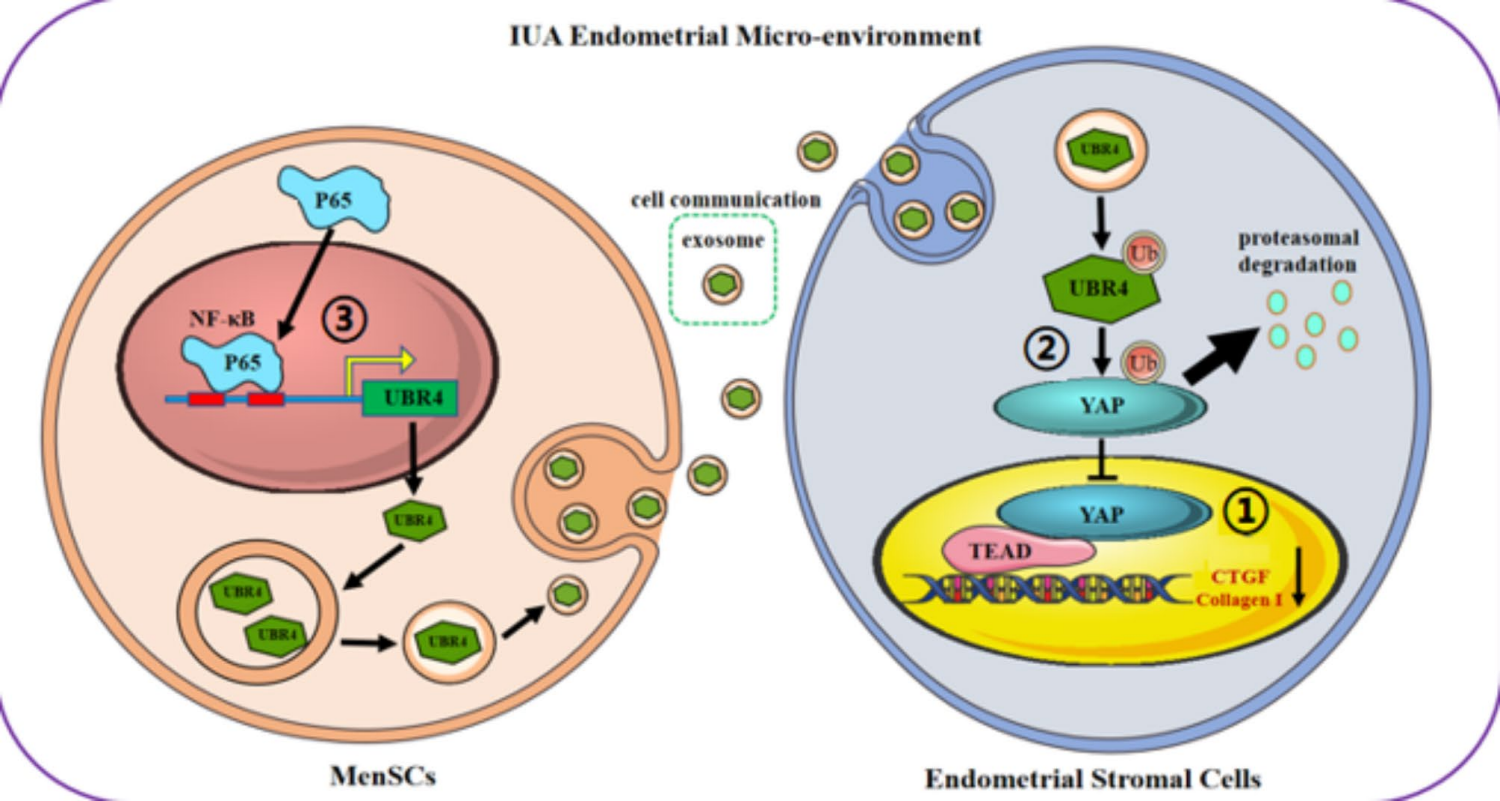
The mechanism graph of the regulatory network. A proposed hypothesis for UBR4 in MenSCs-EXO regulated by P65 attenuated endometrial fibrosis through YAP ubiquitination and degradation

Finally, We attempted to improve the efficiency of MenSCs-EXO therapy by modifying MenSCs to increase the UBR4 content in MenSCs-EXO. Our previous study found that PRP or PDGFBB pretreatment enhanced the biological function of MenSCs by activating the NF-κB pathway [52]. NF-κB signaling pathway had an important regulatory role in both tissue injury and repair [33, 34]. Similarly, the NF-κB signaling pathway also exerted a regulatory effect on human mesenchymal stem cells. A study by Azita Asadi et al. found that apigenin could restore the osteogenic differentiation of adipose stem cells in an inflammatory environment by regulating the expression of some key factors in the NF-κB signaling pathway [53]. In hematopoietic stem cells, the NF-κB signaling pathway was activated, which enhanced the survival and function of hematopoietic stem cells, to promote the recovery of myeloid differentiation function and hematopoietic function [37] We also predicted that P65, a transcription factor in the NF-κB family, could bind to the UBR4 promoter region, thus we hypothesized that P65 could promote the expression of UBR4 in MenSCs. It was found that P65 would transfer to the nucleus after activation and then drive the transcription of downstream target genes by binding to the recognition site in the promoter of the target genes [54]. Cytoplasmic/nuclear localization of P65 might also affect the ability of MSCs. Nuclear translocation of P65 was involved in regulating the osteogenic differentiation of MSCs as well as the odontogenesis of dental pulp stem cells [55, 56]. Our results revealed a significant nuclear translocation of P65 after P65 overexpression, which set the foundation for the subsequent regulation of UBR4 transcription. UBR4 expression increased and decreased when P65 expression was upregulated or downregulated, and Dual-luciferase reporter and ChIP assays confirmed that P65 could bind to the UBR4 promoter region. These results suggested that P65 could promote the expression of UBR4 by binding to the UBR4 promoter region. Combined with our findings, we suggested that P65 transcriptionally regulating the UBR4-mediated degradation of YAP ubiquitination was the potential molecular mechanism for MenSCs-EXO treatment of endometrial fibrosis (Fig. 7).

## Conclusion

In conclusion, we clarified that UBR4 was the key molecule in the repair of IUA by MenSCs-EXO and alleviated endometrial fibrosis by boosting YAP ubiquitination degradation. Moreover, we also demonstrated the molecular mechanism of P65 transcriptional activation regulating UBR4 expression in MenSCs, which laid the theoretical foundation for the clinical application of MenSCs-EXO in the treatment of IUA.

## Methods

### Isolation and culture of MenSCs and EndoSCs in vitro

MenSCs were isolated from menstrual blood collected from IUA patients and healthy women with written informed consent. MenSCs were isolated and cultured using the same methods as our previous studies [18]. Briefly, menstrual blood samples were collected the day after the onset of menstruation. Then the samples were separated through density gradient centrifugation. The cells in the middle layer were carefully transferred to another 15ml centrifuge tube, then they were cleaned with phosphate buffered saline (PBS), and finally, we planted cells in a 60 mm tissue culture dish (NEST). The cells were cultured in DMEM/F12 medium (HyClone, Logan, UT, USA) added with 10% fetal bovine serum (Gibco, Waltham, MA, USA) and 1% penicillin-streptomycin (PS; Sigma-Aldrich), and were incubated in an environment containing 5% CO2 at 37℃. When the cell density reached 80–90%, cell passage was performed with 0.25% trypsin (Sigma-Aldrich). The fresh culture medium was replaced after 24 h of isolation, and the medium was changed every 2 days. The third to the sixth generation MenSCs were used for the following experiments.

We isolated EndoSCs from the endometrial sample of IUA patients who underwent hysteroscopic adhesiolysis in Shengjing Hospital Affiliated of China Medical University. The endometrial tissues were chopped and added with type I collagenase (Solarbio, Jiangsu, China) and left for 30 min at room temperature. The EndoSCs were separated by a 40 μm filter (Biofil, Guangzhou, China) and were centrifugated with 250×g at room temperature for 20 min. Subsequently, the suspended cells were centrifuged at room temperature at 2000 g for 30 min, and the middle layer cells were collected. EndoSCs were cultured in DMEM/F12 medium supplemented with 10% fetal bovine serum at 37℃ and 5% carbon dioxide (CO2).

### Lentivirus constructs and transfection

The UBR4 and P65 knock-down Lentivirus, P65 over-expression Lentivirus, and control lentivirus were designed and purchased from HANBIO (Shanghai, China) and the sequences are shown in Table 1. All lentivirus transfection procedures were carried out according to HANBIO’s protocol. Transfection MOI of UBR4 and P65 knock-down Lentivirus and P65 over-expression Lentivirus were 30, 30, and 50 respectively. The fresh culture medium was replaced 24 h after transfection. The cells were collected 72 h after lentivirus transfection to examine knock-down and over-expression efficiency.

**Table 1:** The sequence of Sh-UBR4, Sh-P65 and negative control

Lentivirus	Sequence
Sh-UBR4-673	GCGCUACGUUGUCCUGGAUTT
Sh-UBR4-3493	GCAACTCATTGACACCTAT
Sh-UBR4-11395	GCGUCCACAAGGAGCAUGATT
Sh-P65-1	CCTGTCCTTTCTCATCCCATCTTTG
Sh-P65-2	GGGATGAGATCTTCCTACTGTGTGA
Sh-P65-3	CAGATACAGACGATCGTCACCGGAT
ADV-NC	TTCTCCGAACGTGTCACGTAA

### Isolation of MenSCs-EXO

P3-P6 MenSCs with or without knocking down UBR4 were selected to obtain exosomes with or without knocking down UBR4 (EXO^UBR4−^ or EXO). The serum components in the medium were fully removed after repeated washing with PBS more than 3 times.MenSCs were starved with serum-free DMEM/F12 and cultured for 24 h at 37℃ and 5% CO2. The serum-free culture medium of MenSCs was collected and centrifugated with 2000×g at 4℃ for 20 min to remove the cell debris and dead cells. The MenSCs supernatant was filtered by a 0.22 μm filter (Millipore, Carrigwohill, County Cork, Ireland) to remove the large vesicles in the supernatant. In this study, exosomes were isolated by ultrafast centrifugation. The ultracentrifugation procedure was set as ①10,000 ×g 1 h, ②100,000 ×g 4 h, and the temperature condition was 4℃. The precipitate was resuspended with 100 µl PBS.

### RNA extraction, reverse transcription, and quantitative real-time PCR (RT-PCR) analysis

Total cell RNA was extracted by trizol following the manufacturer’s instructions. Then 1 µg RNA is reverse-transcribed into 20 µl complementary DNA (cDNA) using a reverse transcription system (R323-01, Vazyme) and qRT-PCR was performed with ChamQ Universal SYBR qPCR master mix (Q711-02, Vazyme) in 7500 software v2.0.6 (Life Technologies, Carlsbad, CA, USA). The primers used in this study were designed and synthesized from Sangon Biotech Co, Ltd. (Shanghai, China) and were listed in Table 2. The gene expression change was analyzed via 2 ^−ΔΔCt^ with GAPDH as an internal reference, and each RNA sample was repeated three times.

**Table 2:** The sequence of primer for quantitative real-time PCR

Gene name	Sequences
*UBR4*	F: GACGAGACCATGGTTGAACTAG R: GAAGGGTAGAACCATCTGTCG
*P65*	F:TGTGAAGAAGCGGGACCTGGAG R:AAGCAGAGCCGCACAGCATTC
*GAPDH*	F:TGACATCAAGAAGGTGGTGAAGCAG R: GTGTCGCTGTTGAAGTCAGAGGAG

*Protein extraction and Western Blot*: Proteins were lysed and extracted from MenSCs cells, EndoSCs cells, and rat uterine tissue using freshly prepared cell lysis RIPA (Solarbio, Jiangsu, China) containing 1% protease inhibitor PMSF (Solarbio, Jiangsu, China) Protein concentrations were measured with a BCA kit (Beyotime Biotechnology, Shanghai, China). The proteins went through SDS-PAGE gel (Beyotime Biotechnology, Shanghai, China) to be separated and were transferred to a PVDF membrane (Millipore, USA), then were blocked by 5% defatted milk powder to prevent nonspecific binding of PVDF membrane and the primary antibody. Next the PVDF membrane was incubated with UBR4 (1:500, ab86438, Abcam, USA), β-tubulin (1:3000, ab179513, Abcam, USA), CD81 (1:1000, ab79559, Abcam, USA), COL I (1:2000, ab34710, Abcam, USA), YAP (1:2000, #14,074, CST, USA), CTGF (1:1000, ab6992, Abcam, USA), P65 (1:1000, #8242, CST, USA), Ubiquitin (1:1000, ab134953, Abcam, USA) and GAPDH (1:1000, #5174, CST, USA) together at 4 °C overnight. The next day, the membrane was incubated with Goat Anti-Rabbit IgG H&L (HRP) (1:3000, ab6721, Abcam, USA) or Anti-Mouset IgG H&L (HRP) (1:3000, ab6789, Abcam, USA) together at room temperature for 2 h. The bands were visualized with ECL solution (#1,862,420, Thermo Fisher Scientific, USA). The gray value was calculated by ImageJ software.

*Immunofluorescence*: P3 to P6 EndoSCs and MenSCs transfected P65 over-expression Lentivirus or control lentivirus were implanted on cell coverslips (14 mm, NEST). P3 to P6 EndoSCs cells from IUA patients were treated with PBS, 100ng/ml EXO^UBR4−^ and EXO for different groups for 24 h. The cell coverslips were washed twice with PBS, then fixed with 4% paraformaldehyde at room temperature for 30 min and blocked with 10% goat serum (ZLI-9022, ZSGB-Bio, Beijing, China)for half an hour at 37 °C. The sections of the IUA rat uterus were dewaxed, transparent, rehydrated, and repaired by the thermal repair method. The sections were immersed in 3% hydrogen peroxide solution at 37 °C for 30 min and then were blocked with 10% goat serum at 37℃ for 30 min. The cell coverslips and sections of IUA rat uterus were incubated with UBR4 (1:500, ab86438, Abcam, USA), Ki67 (1:100, ab15580, Abcam, USA), COL I (1:100, ab34710, Abcam, USA), YAP (1:100, #14,074, CST, USA), CTGF (1:100, ab6992, Abcam, USA) and P65 (1:100, #8242, CST, USA) together at 4 °C overnight. The next day the cell coverslips and sections were washed three times with PBS, followed by incubating with secondary antibodies (1:500, A0562, A0516, FITC-labeled goat anti-rabbit IgG, Cy3-labeled goat anti-rabbit IgG, Beyotime, Beijing, China) together at room temperature for 2 h. The cell coverslips were washed with PBS three times and were incubated with DAPI (1:20, C1005, Beyotime Biotechnology, Shanghai, China) for 5 min, and sealed with fluorescent quench agent or neutral resin. The fluorescence intensity was observed under A Nikon Eclipse Ni (Nikon, USA).

*IUA rat model establishment and treatment*: SPF 10-week-old female SD rats were purchased from HFK Bioscience (Beijing, China). They were kept in the SPF Animal Department of Shengjing Hospital affiliated of China Medical University, with appropriate and constant temperature and humidity and a light/dark time ratio of 14 h/10 h. The estrous female mice were anesthetized with 1ml/kg 3% sodium pentobarbital. Under aseptic operation conditions, the abdominal wall was cut layer by layer, and bilateral uterine horns were fully exposed. A small incision was made near the fallopian tube of the uterus. The improved 16G syringe needle was inserted into the uterus and scratched and scraped the uterine wall several times (about 2/3 of the uterine wall in depth) until the blood came out. The uterine wall incision was closed after sterile saline flushing of the uterine cavity. After the IUA rat model establishment, the rats were randomly divided into 3 groups: IUA group, EXO^UBR4−^ group, and EXO group, with n = 6 in each group. After 7 days of operation, rats in each group were anesthetized to open the abdominal wall and expose the uterus. PBS or exosomes were injected with a 32G syringe at multiple points along the long axis of the bilateral uterus starting from the uterine horn. Each rat in the EXO^UBR4−^ group and EXO group was injected with 100 µl exosome suspension (100ng/ml), and each rat in the PBS group was injected with the same volume of sterile PBS. After 7 days of feeding, the rats in each group were sacrificed under anesthesia for sample collection.

*H&E staining and Masson staining*: The samples of IUA rats were cut into small sections and immersed in 4% paraformaldehyde for 48 h. The fixed uterine tissue was dehydrated, transparent, embedded in paraffin, and sliced into 5 μm paraffin sections. The sections were dewaxed, transparent, rehydrated, and stained with hematoxylin and eosin (H&E). Endometrial thickness was measured in 3 randomly selected directions for each section, and the mean value was calculated as the endometrial thickness of this section. The number of glands was counted by eyes. The fibrosis area of endometrium was observed by Masson staining (#G1340, Solarbio, Jiangsu, China), and the percentage of fibrosis area in the total area was calculated by ImageJ.

*Immunohistochemistry*: The procedure before staining was the same as immunofluorescence.Then the sections were incubated with COL I (1:2000, ab34710, Abcam, USA), CTGF (1:1000, ab6992, Abcam, USA) and CD31 (1:1000, ab28364, Abcam, USA) at 4 °C overnight.The next day, the sections were washed twice with PBS, incubated with the secondary antibody at 37 °C for 30 min, and washed twice with PBS for 5 min each time. Then the sections were stained with 3,3-diaminobenzidine (DAB) and Mayer’s hematoxylin. The percentage of cells with different staining intensities was calculated by ImageJ, and staining intensity was divided into four levels: 0 for negative, 1 for weakly positive, 2 for medium positive, and 3 for strong positive.

*Chromatin immunoprecipitation (ChIP)*: P3 to P6 MenSCs were cultured in a 10 cm diameter culture dish with a density of 10^6^ cells and with 8 culture dishes in each group respectively according to the instructions of the ChIP kit (#9004, CST, USA). After the cells were cross-linked with formaldehyde, the cells were broken with an ultrasonic crusher, and the P65 antibody (#8242, CST, USA) antibodies were added and mixed overnight at 4℃.On the second day, protein G magnetic beads were added to collect the antibody-bound chromatin DNA complex. Then the DNA was washed and unlinked from protein-DNA cross-linking and treated with protease K to obtain purified DNA. The 2µL DNA was used for real-time PCR to detect the immunoprecipitated DNA of the target antibody. The qRT-PCR reaction system was 2 µl DNA, 10µL SYBR, 1 µl upstream and downstream primers (upstream: AGTGCCTTGATCTCGACTCAC; downstream: GTGGTAGCACACTCCTGGAG) respectively, and 6 µl DEPC water. Amplification conditions: (a) pre-denaturation at 95℃for 5 min; (b) denaturation at 95℃for 30s; (c) renaturation at 62℃for 30s and d.extension at 72℃for 30s, a total of 34 cycles for b-d.

*Dual-luciferase reporter assay*: To predict the target binding site of P65 in the UBR4 promoter region based on the Jaspar database (http://jaspar.genereg.net/), we constructed the UBR4 wild-type (WT) and mutant (Mut) recombinant luciferase reporter vector (Ribobio, Guangzhou, China). The reporter plasmid and PEX3-P65 plasmid or PEX3-basic vector (GenePharma, Shanghai, China) were transferred into 293T cells together. The cells were cultured after transfection for 48 h, then 50 µl PLB passive lysis solution was added and left for 15 min at room temperature for cell lysis. The cell lysates were collected, and the 20 µl lysates were mixed with and 100 µl luciferase substrate for each group. The firefly luciferase values were detected using a bioluminescence detector, and the experimental results were recorded. Adding 100 µl termination solution, the renilla luciferase value was detected. Dual-Luciferase Reporter Assay Kit was used to perform Dual-luciferase reporter assay (E1910, Promega, Madison, USA). The expression degree of the target gene is equal to the value of firefly luciferase/ the value of renilla luciferase.

### Co-immunoprecipitation (Co-IP) assay

Four hours before the EndoSCs collection, the final concentration of proteasome inhibitor MG-132 (dimethyl sulfoxide) was added and the culture medium was washed twice with 1×PBS. Then lysis solution was added to lysate cells on the ice for 30 min. Scrape off the lysed cells with a pre-cooled cell scraper and transfer them to a clean 1.5 mL EP tube. Centrifuge at 12000r/min at 4℃ for 15 min. YAP antibody (#14,074, CST, USA) was added to immunoprecipitate protein at 4 °C overnight. Then Protein A + G Agarose beads were added at 4 °C for 12 h. The precipitation was eluted with elution buffer and was denatured at 100 °C for 5 min to perform the subsequent Western Blot described above.

### Statistical analysis

Statistical software GraphPad Prism 8 (San Diego, CA, USA) was used to process all data and the measurement data was expressed as mean ± standard deviation. Student’s *t*-test was used for the comparison of two sample means, and one-way ANOVA analysis was used for the comparison of multiple sample means. Take P < 0. 05 indicates a statistically significant difference (*P < 0.05, **P < 0.01, ***P < 0.001).

## Data Availability

Data will be made available on request.
